# Health Journalism in Nepal: Evolution, Current Developments and Future Directions

**DOI:** 10.31729/jnma.8850

**Published:** 2024-12-31

**Authors:** Shalik Ram Dhital, Madhu Koirala Dhital, Padam Raj Joshi, Atul Mishra, Bhakta Bahadur KC

**Affiliations:** 1 Health Promotion and Education Association Nepal, Kathmandu, Nepal; 2 Maroba Caring Community, Newcastle, Australia; 3 Health Today Magazine, Kathmandu, Nepal; 4 Nepal Health Press, Baneshwor, Kathmandu, Nepal; 5 National Health Education, Information and Communication Center, Teku, Kathmandu, Nepal

**Keywords:** *development*, *future direction*, *health journalism*, *media*, *Nepal*

## Abstract

Until the late of 20^th^ century, health journalism in Nepal was limited, with information primarily shared orally by traditional healers, Vaidya and physicians. However, the rise of online and digital media has transformed the field, making it multifaceted and enabling faster, broader dissemination of health information through the internet, social media, and multimedia platforms. Since 2006/07, health journalism has gained importance, with the Health Journalist Forum Nepal, established in 2001, playing a significant role. Today, health journalism in Nepal uses print, radio, television, and digital media, with collaboration and authenticity in health reporting. The objective of this viewpoint is to explore historical development, current status, and future directions of health journalism in Nepal.

## INTRODUCTION

Health journalism is an interdisciplinary field which connects the public with the scientific community, translating complex health information into accessible content to raise awareness of global health issues and influences the health behaviour.^1,2^ In Nepal, health journalists function as a watchdog for quality health care delivery and policy formulation, holding politicians and policy makers accountable while ensuring accurate and authentic health reporting. This field involves disseminating health information through mass media, covering diverse topics such as news, scientific research, health policies, and the evaluation of health programs. Health journalism plays a critical role in holding institutions accountable, influencing health policies, and educating people about health issues.^3^ Although few journalists have specialized training in health journalism, many of them report various health issues across the country. This paper aims to explore historical development, current status, and future directions of health journalism in Nepal.

## EVOLUTION OF HEALTH JOURNALISM

In ancient times, health information was disseminated verbally or through texts by traditional healers, physicians, and Vaidya in the Indian continent. Modern health journalism practices in India grew with the development of newspapers and health magazines in the late 20^th^ century and have since slowly become digitalized.^4^ In the 20^th^ century, the United States saw the rise of health sections in major newspapers and magazines.^5^ With the advent of television and the internet, health journalism expanded to include multimedia platforms, addressing issues from medical breakthroughs to public health crises.^6^ In Australia, health journalism has developed from early newspaper health columns to comprehensive reporting, including the use of digital platforms and multimedia.^7^

In Nepal, the Gorkhapatra Newspaper was the first ever media portal, starting from 1901/04/07 AD, and it released health-related information focusing on Kala-azar, malaria, and other communicable diseases prevention. Radio Nepal was established in 1951 AD, and Nepal Television in 1985 AD, with many private television stations broadcasting health messages to the public. After the people's movement in 1989/90 AD, democracy was established, and Nepal's constitution (1990) provided significant space for health journalism in the media. This covered guaranteed press freedom, with the media ranking 4th component of state. The broadsheet daily newspaper started, providing a separate platform for health issues. The Sadhana health magazine played a significant role in health journalism, starting in 1991 AD. The Kantipur Daily Newspaper also began writing health bit in 1992 AD recruiting a separate health reporter. Gradually, Nepal Samachar Patra, Rajdhani, Space Times, Annapurna, Naya Patrika, and Nagarik became dedicated to disseminating health messages and allocated their separate health reporters. Another health magazine called "Capsule Digest" existed, but it was not publicly sold to the public. The Health Journalist Society was established in 2001 AD, and the Nepal Media Development Centre organized health journalism training in Nepal. The Health Journalist Group (HJG), established in 2010 AD, contributed to the development of health journalism, particularly in disseminating reproductive health and Human Immuno-deficiency Virus / Acquired Immuno-deficiency Syndrome (HIV/AIDS) issues. Health journalism gained prominence after the people's movement of 2006/07 followed by adopting one health approach in Nepal.^8^ The field has expanded despite limited health training opportunities, focusing on multi-level health issues. The Health Journalist Forum (HJF) Nepal, established in 2014 AD. Both HJF and HJG aimed to enhance health reporting, raise health awareness and diseases prevention reporting (Table 1).

**Table 1:** Major online Media for Health Journalism in Nepal.

SN	Name	Year	Health Coverage
1	Onlinekhabar	2006	Health news, policy updates, and public health issues.
2	Setopati	2008	Health policies, medical research, and health features.
3	My Republican	2009	Health news, medical updates, and health features
4	Nepal Health News	2012	Delivering health-related news
5	Ratopati	2014	Social health issues.
6	Health Today Nepal	2017	Health information, expert interviews, and promotion.
7	Mahila Swasthya	2017	Issues related to women's health and well-being
8	Nepali Health	2018	General health newss specifically reproductive health and nutrition related information and experts' opinion.
9	Health TV online	2018	Health news, tips, education, and expert interviews.
10	Swasthya khabar	2019	Health information, expert interviews, and promotion.
11	Health Pati	2019	Medical research, wellness tips, disease prevention, and policies.
12	Health Aawaj	2019	Health tips and news, medical updates.
13	Swasthya Samachar	2019	Health updates and information provided regionally.
14	Swasthya Page	2020	Health updates and information provided regionally.
15	Medical Patra	2020	Health tips, information about diseases prevention and healthcare practices.
16	Healthy Khabar	2020	Disease prevention, health tips, and healthcare policies.
17	Health News Nepal	2020	Medical innovations, health tips, disease prevention, and healthcare policies.
18	Swasthya Diary	2022	Health information dissemination.
19	Nepal Health Press	2022	Medical research, disease prevention, wellness tips, and updates on health policies, and health information dissemination to people.
20	Health Bani	2022	News updates, health information, wellness tips, and insights into healthcare practices.
21	Swasthya Darpan News, Chitwan	2022	Health updates and information provided regionally.
22	Health Patra	2024	Health news and tips, medical advice, and information on disease prevention.

**Table 2:** Major Print Media for Health Journalism in Nepal.

SN	Name	Year	Health Coverage
1	Gorkha Patra	1959	National and local health issues, public awareness.
2	Sadhana	1992	Offering information and updates on various aspects of health and wellness.
3	The Kathmandu Post	1993	Health news, health policy updates, and features on medical research and health issues.
4	The Kantipur	1993	Health news, interviews, and health education.
5	The Himalayan Times	2001	Health news, medical research, health policies, and health-related features.
6	Nagarik daily	2008	Health News, and expert articles
7	Swasthya khabar Patrika	2010	Health news, medical innovations, and policies.
8	Janswasthya Sarokar	2010	Health update, information about diseases prevention and control, lifestyles, nutrition, corporate health, health news and physician consultation.
9	Nepal Health Journal	2011	Health information, medical research, and health policy.
10	Health Today Nepal	2014	Medical advice, health trends, disease prevention, and wellness tips.

**Table 3:** Major audio- and audio-visual Media for Health Journalism in Nepal

SN	Name	Year	Health Coverage
1	Radio Nepal	1951	Health news, diseases prevention, health campaigns, and education.
2	Nepal Television	1985	Health and documentaries, and public health debates.
3	Kantipur FM	1998	Health news and education, disease prevention, interviews.
4	Community Radios	2000	Health issues, health education, disease prevention, and community health events.
5	Kantipur Television	2003	Health news, features on health issues, and policies.
6	Global TV	2011	Expert opinion through interviews.
7	AP1 Television	2012	Health updates, health documentaries, and talk shows.

Through professional journalism. It collaborates with the government of Nepal and the Ministry of Health and Population (MoHP) for authentic reporting. Health journalists in Nepal have increased in recent times covering many health issues.^9^ Similarly, the Nepal Health Care Journalist Society (HCJS) is active in providing training, interactive sessions, and workshops for upgrading health journalism in Nepal among health journalists throughout the country.

## CURRENT SITUATION OF HEALTH JOURNALISM IN NEPAL

Previously, health journalism was a part of normal journalism, nevertheless now it is growing separately as a distinct field within the media mainstream. The scope of health journalism has increased significantly, and it played positive roles during various outbreaks and public health emergencies, such as dengue, cholera, Japanese encephalitis etc. Health journalists have played a significant role in the containment of coronavirus disease (COVID-19) through public awareness and Risk Communication and Community Engagement efforts. They also report remote health issues and typical cases.^10^ The joint initiation was taken by the HJF and HCJS , in developing health journalism as a profession in Nepal. Approximately 10% of total messages were health-related, but after the COVID-19 pandemic, this ratio has increased significantly, making health reporting become a priority in Nepal. Currently, online media has become popular, with more than 20 channels from Kathmandu and major cities outside Kathmandu becoming famous for health.

Between 40 and 50 health beats and a few news portals are active in disseminating health messages, and the major print media are listed (Table 2).

Similarly, audio and audio-visual source of health information is disseminating through radio, FMs and television, and some media are presented (Table 3).

The establishment of health journalism in Nepal is becoming a part of health literacy and health policy through partnerships with the GON, the MoHP, and National Health Education, Information and Communication Center (NHEICC), external development partners (EDPs), and the communities. A team of health journalists in collaboration with the Nepal Medical Association (NMA) and the World Health Organization (WHO) prepared and approved a curriculum on health journalism training. With the help of WHO and NMA, they plan to roll out health journalism training across the country in the future.

## ROLE OF HEALTH JOURNALISM WITHIN A CONTEXT OF NEPAL

Health journalism is an emerging field, focused on health-related issues across the country. This specialized approach aims to fulfill the gap by placing the "right journalists in right scope". Health Journalism became popular during disaster and crises in the country such as earthquake, COVID-19, and flooding due to its significant role in shaping public perception and influencing decision on public health issues.

This discipline serves multiple purposes, including promoting health, providing the public with accurate information, educating communities, and lobbying for policy changes. It raises awareness about disease prevention, healthy lifestyles, and essential healthcare issues. Health journalism also highlights disparities in healthcare, especially in rural areas, and advocates for equitable access to health and medical services for poor and marginalized communities.

During health crises, such as epidemics or pandemics, health journalists provide timely updates and information on prevention, management and treatment. They hold healthcare institutions accountable by investigating issues like corruption, inefficiency, and inadequate access to services. Additionally, health journalism supports national health campaigns and promotes mental health awareness- an important but often overlooked issue in Nepal.

Furthermore, health journalism helps build public trust in healthcare systems by offering reliable information. It also informs the public about global health trends and research, influencing national and subnational health practices and contributing to informed decisionmaking. In Nepal, health journalism informs, motivates, educates, advocates, and supports improvements in healthcare, contributing to the country's overall wellbeing. The core functions of health journalism can be categorized into four key areas:(1) Interpretative: examining, communicating and simplifying the complex health and medical information, (2) Adversarial: institutional accountable, exposing malpractices, support in investigating corruption and irregularities, (3) Disseminating: delivering accurate, relevant health messages in time for research, policy and practices, and (4) Populist mobilizer: through community engagement for health awareness.^11^

In Nepal, many health journalists collaborate with NHEICC, a national focal body of health communication to enhance reach and impact of their work.

## CHALLENGES AND OPPORTUNITIES

Health journalism in Nepal faces several challenges, including limited training opportunities for journalists, difficulties in accessing reliable health data, and the low commercial viability of health-focused media. These challenges can hinder journalists' ability to deliver accurate, timely and engaging health information to the public. However, there are also significant opportunities to overcome these issues. Health journalists should adopt modern platforms like podcasts, video documentaries, and data visualization tools to engage a wider audience and present complex health issues in an accessible format. By leveraging scientific data and collaborating with health experts and academic institutions, health journalists can improve the credibility and reliability of their work. Expanding the use of social media platforms and fostering community engagement can also enhance local health communication, ensuring that information reaches diverse populations. These approaches not only help overcome barriers but also contribute to building a more informed public, equipped with authentic, evidence-based health information.

## WAY FORWARD

Looking ahead, efforts should focus on further capacity building for health journalists, strengthening partnerships between media outlets and health organizations, and promoting interdisciplinary collaboration. Additionally, incorporating health promotion approaches and behavior change communication strategies into health reporting will be crucial for improving public health outcomes. By embracing innovation and investing in advanced training and resources, health journalism in Nepal can become a transformative force, raising in health awareness and encouraging positive health behaviors across the diverse communities.
